# Burden of Antimicrobial Resistance Among Women with Post-Partum Infections in Low-Middle Income Countries: A Systematic Review

**DOI:** 10.1007/s44197-024-00222-8

**Published:** 2024-04-17

**Authors:** Caterina Monari, Lorenzo Onorato, Nicola Coppola, Mario C. B. Raviglione, Giorgia Gon

**Affiliations:** 1 Infectious Diseases Unit, AOU Luigi Vanvitelli, Naples, Italy; 2 Section of Infectious Diseases Unit, Department of Mental Health and Public Medicine, University of Campania Luigi Vanvitelli, Via L. Armanni 5, 80131 Naples, Italy; 3 MACH Centre, University of Milan, Milan, Italy; 4 Department of Infectious Disease Epidemiology, London School Hygiene and Tropical Medicine, London, UK

**Keywords:** MRSA, ESBL, MDRO, Low-middle-income countries

## Abstract

**Background:**

Due to the rising incidence of multidrug-resistant (MDR) pathogens, especially in Low-Middle-Income Countries (LMIC), post-partum infections represent a significant treatment challenge.

**Methods:**

We performed a systematic review of the literature from January 2005 to February 2023 to quantify the frequency of maternal post-partum infections due to MDR pathogens in LMICs, focusing on methicillin-resistant *Staphylococcus aureus* (MRSA) and/or extended-spectrum beta-lactamase (ESBL)-producing *Enterobacterales*. Secondary objectives: description of antimicrobials’ prescriptions.

**Findings:**

We included 22 studies with 14,804 total bacterial isolates from 12 countries, mostly from WHO African-Region. Twelve papers described wound- and 10 puerperal-infections. Seven were high-quality articles. Seventeen studies reported data on MRSA, and 18 on ESBL-producing *Enterobacterales*. Among high-quality studies, MRSA ranged from 9.8% in Ghana to 91.2% in Uganda; ESBL-producing *Enterobacterales* ranged from 22.8% in Ukraine to 95.2% in Uganda. Nine articles, mostly on C-sections, described different protocols for antibiotic prophylaxis and/or post-partum treatment.

**Interpretation:**

We described a high burden of post-partum infections caused by MRSA and/or ESBL-producing *Enterobacterales* in LMICs, but only a few studies met quality standards. There is an urgent need for high-quality studies to better describe the real burden of antimicrobial resistance in low-resource settings and inform policies to contain the spread of multidrug-resistant organisms.

**Supplementary Information:**

The online version contains supplementary material available at 10.1007/s44197-024-00222-8.

## Introduction

Infections are an important cause of maternal mortality and morbidity worldwide [1, 2]. Post-partum infections, defined by the World Health Organization (WHO) as “infections of the genital tract and surrounding tissues from labour onset or rupture of membranes until 42 days postpartum”, represent a significant, and often preventable, healthcare burden [3]. The most common post-partum infections include endometritis (or puerperal sepsis), urinary tract infections (UTI), bloodstream infections (BSI), and surgical site infections (SSI) [4]. WHO estimates that direct obstetric infections, including maternal sepsis, are the third most common cause of maternal mortality, representing 10.7% (95% Uncertainty Intervals, UI, 5.9–18.6%) of all deaths worldwide [1]. More in details, puerperal infectious morbidity affects 5–10% of pregnant women [5]. The burden of maternal deaths due to infections is higher in low-middle income countries (LMICs) compared to high-income countries (HICs) (10.7% *versus* 4.7%), with southern Asia and sub-Saharan Africa together accounting for 83.8% of all maternal deaths (13.7% in Southern Asia and 10.3% in Sub-Saharan Africa) [1].

Due to the rising misuse and overuse of antimicrobials, post-partum infections are a significant therapeutic challenge, because they are caused by an alarmingly increasing rate of pathogens resistant to the commonly used antibiotics [4]. Antimicrobial Resistance (AMR) represents one of the main Global Health threats of the twenty-first century. A recent predictive statistical model by Murray et al*.* estimated 4.95 million deaths globally attributable to bacterial AMR in 2019 [6]. Moreover, it is estimated that, if no appropriate measures are taken, AMR will cost approximately 10 million lives and US$ 10 trillion per year by 2050 [7].

In 2015 the WHO endorsed a global action plan on AMR to improve awareness, strengthen surveillance, reduce the incidence of infections, optimize antimicrobials use, and ensure sustainable investments in countering AMR [8]. In addition, in 2017  the WHO published a list of antibiotic-resistant “priority pathogens”, to guide and promote research and development of new antibiotics. Nevertheless, worrying levels of resistance have been reported in all countries, but with a disproportionately higher burden in LMICs [6, 9], with the result that common diseases are becoming untreatable [10]. The last GLASS (Global Antimicrobial Resistance and Use Surveillance System) report described, in LMICs compared to HICs, a concerning higher rate of *Escherichia coli* resistant to 3rd generation cephalosporins (3GC) (58.3% *vs* 17.5%), and methicillin-resistant *Staphylococcus aureus* (MRSA) (33.3% *vs* 15%) [11].

Reducing maternal mortality and tackling AMR are global health priorities and a target of the 2015–2030 Agenda for Sustainable Development [12, 13]. In 2020 two new AMR indicators were included in the monitoring framework of the Sustainable Development Goals (SDGs) within the target 3.d, *i.e.*,  to monitor the frequency of BSI due to 3GC-resistant (3GCR) *Escherichia coli* and MRSA [11, 14].

This systematic review aims to describe and quantify the burden of infections due to multidrug-resistant (MDR) pathogens among women in the peri-/post-partum period in LMICs. Moreover, since *Staphylococcus aureus* and *Enterobacterales*, mainly *Escherichia coli* and *Klebsiella spp.*, are the most common causative agents of post-partum infections, and MRSA and ESBL-producing *Enterobacterales* belong to the “high” and the “critical” priority list of antibiotic-resistant pathogens, respectively [15], we focused on infections caused by these pathogens.

## Material and Methods

### Search Strategy

We performed a systematic review of the literature applying the search strategy in three electronic databases (EMBASE, Medline, and Global Health) from January 2005 to February 2023. We decided to start the search in 2005, since it was in 2005 that the World Health Assembly raised the question of AMR and requested to strengthen the WHO’s leadership role in containing AMR by providing technical support to its Member States [16]. The search strategy was designed by two authors (C.M. and G.G.).

The search strategy included the combination of three main domains (Text or Medical Subject Headings, MeSH): “peripartum/puerperal” AND “infection” AND “Antimicrobial Resistance”, restricted to humans. Inclusion and exclusion criteria have been applied to title and abstract first, and then to full-text papers by two authors (C.M. and L.O.). Duplicates were removed by one author (C.M.). Reference lists of eligible articles were manually checked for additional potentially relevant papers by the same authors (C.M. and L.O.). Any discrepancies were resolved by means of discussion or consultation with a third reviewer (G.G.). The complete search strategy is provided in Supplementary Table 1.

The systematic review was conducted according to the Preferred Reporting Items for Systematic Review and Meta-Analyses (PRISMA) guideline [17]. The checklist is available as Supplementary Table 2.

### Outcomes

Our primary outcome was to assess the frequency of infections, with emphasis on those caused by MDR pathogens, among women in the peri-/post-partum period in LMICs. More precisely, we focused on infections due to MRSA and/or ESBL-producing or 3GCR *Enterobacterales*. The secondary outcome included the description of antimicrobials’ prescription, in particular peri-partum antibiotic prophylaxis.

### Inclusion and Exclusion Criteria

We included all papers that met the following criteria:

- studies performed in LMICs,
- studies on women in the peri-/post-partum period reporting an infection in this specific period,
- studies reporting data on AMR and/or MDR pathogens, more precisely: studies reporting data on MRSA or ESBL-producing *Enterobacterales* infections, OR studies reporting infections due to *Staphylococcus aureus* characterized by in vitro resistance to oxacillin or cefoxitin or with *mecA* gene detection, OR studies reporting infections due to *Enterobacterales* resistant to 3GC or with ESBL genes detection,
- observational and experimental design,
- studies published as full text,
- studies published in English and/or French and/or Spanish since these are the main spoken languages in LMICs.

We excluded papers with the following characteristics:

- studies reporting ante-natal infection or infections that emerged before the beginning of labour,
- inability to separate outcomes between pregnancy and labour (*e.g*., not exclusive to labour/puerperium),
- inability to separate colonization from infection,
- inability to separate puerperal infection from broader infection (*e.g*., nosocomial),
- inability to separate infectious outcomes from non-infectious outcomes (*e.g.,* maternal morbidities),
- lack of in vitro oxacillin/cefoxitin susceptibility for *S. aureus* AND 3CG susceptibility for *Enterobacterales* in those papers not reporting the frequency of MRSA or ESBL-producing isolates,
- interim reports on prospective cohorts with incomplete data collection or analysis,
- articles reporting on patient cohorts already included in other studies or duplicate data,
- reviews, meta-analysis, case reports, outbreak investigations,
- grey or unpublished literature, conference, and poster abstracts,
- papers published in languages other than English, French or Spanish,
- studies published before 2005,
- studies not conducted in humans.

### Definitions

We used the following definitions:

- *Puerperal sepsis/infection*: “a bacterial infection of the genital tract or surrounding tissues occurring at any time between the onset of rupture of membranes or labour and the 42nd day post-partum, in which ≥ 2 of the following are present: pelvic pain, fever, abnormal vaginal discharge, abnormal smell/foul odour discharge or delay in uterine involution”, according to the WHO definition [3]. This definition encompasses endometritis, chorioamnionitis, wound or surgical site infections secondary to caesarean section (CS) or episiotomy, and sepsis.
- *SSI (Surgical Site Infection):* infections that occur in the part of the body where the surgery took place, according to the *Centers for Disease Control and Prevention* (CDC) [18].
- *Post-partum infections*: we included in this definition all the above-mentioned infections, and mastitis, occurring between the onset of rupture of membranes or labour and the 42nd day post-partum.
- *Multidrug-resistant (MDR): *in vitro non-susceptibility to at least 1 agent in ≥ three antimicrobial categories, according to Magiorakos et al. [19].
- *Low-middle income countries* (LMICs): according to the new World Bank classification based on Gross National Income (GNI) *per capita* [20].

### Data Extraction and Analysis

Two authors (C.M. and L.O.) independently extracted data from each included study, collecting information on AMR burden in women with post-partum infections in resource-limited settings. Key studies characteristics included location of study, study period, study design, number of subjects enrolled, frequency of infections, type of infections described, diagnostic methods, most frequently isolated bacteria, and data on MRSA and ESBL-producing *Enterobacterales*. When the latter data were not available, two authors (C.M. and L.O.) extrapolated them from the reported antibiotic susceptibility profiles, looking at cefoxitin/oxacillin susceptibility for *S. aureus* strains and ceftriaxone susceptibility for *Enterobacterales.* To retrieve 3GC resistance of *Enterobacterales* two authors (C.M. and L.O.) decided to use ceftriaxone, because it was more frequently reported than other agents in the antibiotic susceptibility profiles of isolated bacteria. When this information was not available, authors (C.M. and L.O.) used ceftazidime or cefotaxime susceptibility.

Key information on the frequency of infections due to MRSA and ESBL-producing *Enterobacterales* were: type of infection, pathogens involved, and specific diagnostic methods for resistance detection.

Lastly, C.M. extracted information on the different schemes of antibiotic prophylaxis/therapy used.

### Quality Assessment Criteria

Two authors (C.M. and L.O.) appraised the quality of each study included in the systematic review according to the criteria described in Table 1. Any discrepancies were resolved through consultation with a third reviewer (G.G. or N.C.). C.M. and L.O. used the quality assessment criteria adopted by Wood et al*.* [20], adapted by Joanna Briggs Institute Criteria, for assessing incidence/prevalence studies [21]. For each criterion, studies were classified as having met or not the criteria (yes/no) or were judged unclear, in case of insufficient data. Those studies meeting all five criteria were assessed as high-quality [22]. Regarding criterion 3, *i.e.,* standard definition for maternal infection, we compared the study definition to the definition recognized by international Agencies, such as the CDC or WHO. We did not consider the criteria met if the definitions did not correspond to the CDC or WHO ones.

**Table 1:** Quality assessment criteria adapted from Joanna Briggs Institute criteria ^2^^1,22^

	Quality assessment criteria	
1	Were study participants representative of the study target population? (appropriate recruitment strategy and sampling)	Selection bias
2	Was data analysis conducted with sufficient coverage of the identified sample? (refusals and loss are small [< 15%] and unlikely to be related to the outcome)	Attrition/missing data
3	Was a clear, standard definition used for maternal infection?	Measurement bias
4	Was infection measured reliably using trained/educated data collectors, appropriate/reliable diagnostic procedures, or reliable forms of retrospective data (clinical records meeting standard definitions)?	Measurement bias
5	Were study subjects and setting described in sufficient detail to determine whether results are comparable with other studies?	Poor characterisation of study population

Regarding criterion 5, we defined different population characteristics according to the type of infection reported (puerperal infection or wound infection): we considered age, place of delivery, antenatal care visits, and whether the delivery was performed in emergency or not for puerperal infections, and age, and scheduled/non-scheduled delivery for wound infections.

## Results

**Fig. 1:**
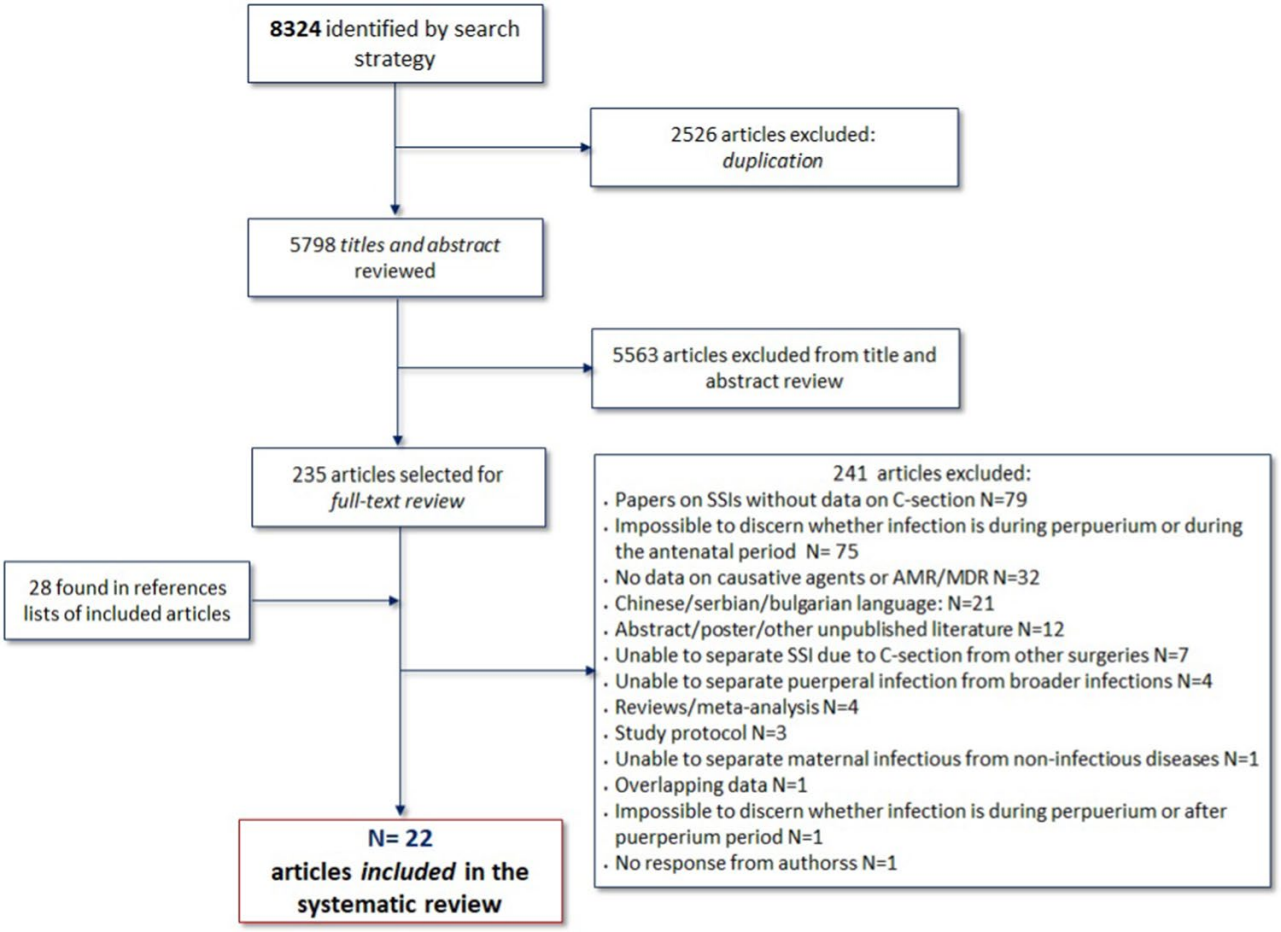
Flow diagram of studies

We identified 8324 potentially relevant articles from searches across EMBASE, Medline, and Global Health databases. Of the total 8324 results, 2526 were duplicates and 5798 were excluded after title and abstract review (Fig. 1). A total of 235 papers were eligible for full-text review. An additional 28 studies were found from reference searching of the eligible papers and were added to the full-text review.

Of the 263 full-text screened papers, 22 were included in the systematic review [23–44]. Main reasons for exclusion after full-text review were: papers on SSIs without data on CS (N = 79, 30.0%), inability to discern whether the infection appeared during pregnancy or puerperium period (N = 75, 28.5%), paucity/lack of information on causative pathogens or AMR/MDR strains (N = 32, 12.2%), and language (N = 21, 8.0%; Chinese N = 19, Serbian N = 1, Bulgarian N = 1). More information is described in the flow diagram of studies (Fig. 1). For one potentially eligible article [45], we contacted the corresponding author because it was unclear from the text whether their outcome fulfilled our inclusion criteria. To date, we have received no response.

### Data and Studies Characteristics

The main characteristics of the 22 papers enrolled in the present systematic review are described in Table 2. Of the total 22 articles included, 21 were in English [24–44], and one was in French [23]. The papers reported data from 12 different countries: 8 countries of the WHO African region (Burkina Faso, Ethiopia, Ghana, Liberia, Nigeria, Rwanda, Uganda, and Tanzania) [23, 24, 26, 27, 30, 31, 34, 36, 40, 41, 43, 44], 2 of the Eastern Mediterranean region (Sudan, Kuwait) [32, 39], 2 of the South East Asian region (Bangladesh, India, Indonesia) [30, 33, 35, 38, 42], and one of the European region (Ukraine) [25, 28, 29, 37].

**Table 2:** Main characteristics of the 22 studies included in the systematic review

	1st Author	Journal, year of publication	Country	Study period	Type of study	Follow-up period, days	N° subjects	n subjects with infection/N (%)
Puerperal infections	Admas [31]	Antimicrobial Resistance and Infection Control, 2020	Ethiopia	January -May 2017	Cross-sectional study	/	166	166
	Ahmed [32]	Annals of Tropical Medicine and Public Health, 2013	Sudan	January 2011- December 2012	Cross-sectional study	/	170	170
	Ahmed [38]	Microbial Drug Resistance, 2014	Bangladesh	November 2010-October 2012	Prospective cohort study	N.A	/	/
	Bebell [24]	PLOS One, 2017	Uganda	March-October 2015	Prospective cohort study	Up to discharge	174	84 (48.2%)
	Kpoto [36]	East African Medical Journal, 2017	Liberia	March–May 2014	Prospective cohort study	120 h after CS	235	49 (21%)
	Ouédraogo [23]	Bull Soc Pathol Exot, 2016	Burkina Faso	February-October 2014	Cross-sectional study	/	7176	102 (1.4%)
	Qadri [33]	International Journal of Current Microbiology and Applied Sciences, 2015	India	April 2013-May 2014	Prospective cohort study	N.A	85	85
	Salmanov (I) [28]	Wiadomości Lekarskie, 2020	Ukraine	January 2015-December 2017	Retrospective multicenter cohort study (14 hospitals)	/	25,344	2460 (9.7%)
	Salmanov (II) [29]	Wiadomości Lekarskie, 2020	Ukraine	January 2015-December 2017	Retrospective multi-center cohort study (7 hospitals)	/	18,427	4172 (22.6%)
	Singh [42]	Journal of Family Medicine and Primary care, 2022	India	April 2019-September 2020	Cross-sectional study	/	2049	106 (5.2%)
SSIs	Alfouzan [39]	Epidemiology and Infection, 2019	Kuwait	2014–2016	Retrospective cohort study	30	7235	152 (2.1)
	De [35]	International Journal of Antibiotics, 2013	India	November 2008-March 2010	Prospective cohort study	30	500	121 (24.2)
	De Nardo [40]	Journal of Hospital Infection, 2016	Tanzania	August-November 2013	Prospective cohort study	30	467	225 (48.2)
	Kifilie [34]	International Journal of Microbiology, 2018	Ethiopia	January-May 2016	Cross-sectional study	/	107	107
	Mpogoro [41]	Antimicrobial Resistance & Infection Control, 2014	Tanzania	October 2011-February 2012	Prospective cohort study	30	345	34 (9.9)
	Njoku [27]	Open Access Macedonian Journal of Medical Sciences, 2019	Nigeria	6 months	Prospective cohort study	30	600	51 (8.5)
	Onuzo [43]	Infection Prevention in Practice, 2022	Ghana	April-July 2017	Prospective cohort study	30	474	61 (12.9)
	Salmanov (III) [37]	Wiadomości Lekarskie, 2021	Ukraine	January 2017-December 2019	Retrospective multicenter cohort study (11 hospitals)	30	2326	342 (14.7)
	Salmanov (IV) [25]	Wiadomości Lekarskie, 2020	Ukraine	January 2017-December 2019	Retrospective multicenter cohort study (7 hospitals)	10	9213	1628 (17)
	Utami [30]	International Journal of Tropical Medicine, 2020	Indonesia	January 2012-October 2016	Retrospective cohort study	N.A	4809	15 (0.3)
	Velin [44]	Annals of Global Health, 2021	Rwanda	September 2019-March 2020	Prospective cohort study	11	795	45 (5.7)
	Wekesa [26]	SAGE Open Medicine, 2020	Uganda	November 2017-April 2018	Cross-sectional study	/	109	109

Sixteen studies were cohort studies (10 prospective, and 6 retrospective) [24, 25, 27–30, 33, 35–41, 43, 44] and 6 cross-sectional studies [23, 26, 31, 32, 34, 42]. Seven papers [23, 24, 28, 29, 31–33] reported data on puerperal infections excluding SSIs, *i.e.*, puerperal sepsis or endometritis, UTI, BSI, and mastitis; 12 papers [24–26, 29, 33, 34, 36, 38–40, 42, 43] evaluated SSIs, *i.e*., infections secondary to CS and episiotomy; 3 [36, 38, 42] reported a composite outcome of puerperal infections and SSIs. Since in the latter 3 papers we could not extract data regarding wound infections only, and most infections evaluated in the studies were not SSIs, we decided to include these papers in the group of puerperal infections. Thus, we divided the 22 studies included in this systematic review into two groups: 10 evaluating puerperal infections (except for SSIs), and 12 evaluating SSIs and episiotomy infections.

Sample size ranged from 85 to 25,344 subjects in studies on puerperal infections, and from 107 to 9213 in studies on wound infections. In 3 studies on puerperal infections [31–33] and 2 studies on wound infections [26, 34], the study sample was represented by all women with a post-partum infection, without reporting information on a denominator for infections.

The characteristics of papers on puerperal infections and wound infections are described in Tables 3 and 4, respectively.

**Table 3:** Characteristics of studies reporting data on puerperal infections (excluding SSIs): type of infection, diagnostic methods, AMR data

Author, year	Denominator, N	Post-partum infections	Post-partum infection/N (%)	Bacterial isolates/N (%)	Diagnostic method	Micro data/ AMR (Y/N)	Pathogens	MDR (reported/extrapolated)
(**a**)								
Admas (2020) [31]	Women with signs/symptoms of puerperal sepsis, N = 166	Puerperal sepsis	/	56/166 (33.7)	Blood culture	Y/Y	*1. S.aureus* *2. E.coli* *3. K.pneumoniae*	Extrapolated MRSA, 3GCR
Ahmed (2013) [32]	Women with puerperal sepsis, N = 170	Puerperal sepsis	/	124/170 (72.9)	Blood culture	Y/Y	*1. S.aureus* *2. C.perfrigens* *3. Listeria mon*	Reported MRSA, no data on ESBL
Ahmed (2014) [38]	Total specimens from women with puerperal infections, N = 676	Puerperal sepsis UTI SSI	/	471/676 (69.7)	Endocervical swab, urine, wound swab	Y/Y	*1. E.coli* *2. S. haemolyticus* *3. Proteus spp.*	Reported MRSA, reported ESBL + *E. coli*, extrapolated ESBL + *Enterobacteriaceae* and *Klebsiella spp*.
Bebell (2017) [24]	Febrile women with puerperal sepsis, N = 174 -193	Endometritis UTIs BSI	76/193 (39) 25/175 (14) 5/185 (3)	/ 23/25(92) 5/5 (100)	Blood and urine culture	Y/Y	*UTI:* *1. Acinetobacter spp.* *2. E.coli* *3.K.pneumoniae, CoNS*	No data on MRSA, reported ESBL
Kpoto (2017) [36]	Total CS, N = 235	Puerperal sepsis (SSI, episiotomy infection, UTIs, breast abscess, fever with purulent lochia)	49/235 (21) Median time 7 days (IQR 6–9), all SSI between 3–22 days post CS	25/49 (51)	Endocervical swab, wound swab, urine culture	Y/Y	*1. S.aureus and E.coli* *2.Pseudomonas aer*	No data on MRSA, reported 3GCR
Ouédraogo (2016) [23]	Total deliveries, N = 7176	Puerperal sepsis	102/7176 (1.4)	61/102 (59.8)	Endocervical, vaginal swab	Y/Y	*1. E.coli* *2. S.aureus* *3.Proteus spp., Streptococcus spp.*	Extrapolated MRSA, ESBL
Qadri (2015) [33]	Women with puerperal sepsis, N = 85	Puerperal sepsis	/	85/85 (100)	Vaginal and cervical swab, fluid from peritoneum or Douglas pouch	Y/Y	*1. GBS* *2. E.coli* *3. S.aureus*	Reported MRSA, ESBL
Salmanov (I) (2020) [28]	Total N of deliveries, N = 25,344	Puerperal sepsis	2460/25344 (9.7)	4879 bacterial strains from 2460 women	Uterine secretion samples	Y/Y	*1. E. coli* *2. E. faecalis* *3. Streptococcus spp.*	Reported MRSA, ESBL
Salmanov (II) (2020) [29]	Total N of breast-feeding women who gave birth < 1 month postpartum, N = 18,427	Mastitis	4172/18427 (22.6)	4758 isolates from 4172 milk samples	Milk samples	Y/Y	*1. S. aureus* *2. E. coli* *3. Enterobacter spp.*	Reported MRSA, extrapolated 3GCR
Singh (2022) [42]	Total N of women hospitalized to obstetrics emergency, N = 2049	Puerperal sepsis (SSI, endometritis, episiotomy infections)	106/2049 (5.2)	54 20 37 18	vaginal swabs pus culture urine culture blood culture	Y/Y	*1. K. aerogenes* *2. E. coli* *3. S. aureus*	No data on MRSA, extrapolated 3GCR

*N.A.* not available, *N* number, *CS* caesarean section, *SSI* surgical site infection, *Y/N* yes/no, *CoNS* Coagulase Negative *Staphylococcus*, *MRSA* methicillin-resistant *Staphylococcus aureus*, *3GCR* 3rd generation cephalosporin resistant, *ESBL* extended-spectrum beta-lactamase

**Table 4:** Characteristics of studies reporting data on SSIs: type of infection, diagnostic methods, AMR data

1st author	Denominator, N	Type of infection	SSI/N° total subjects	Assessment methods n° bacterial isolates/N tot	Micro/ AMR data	Pathogens	MDR (reported/extrapolated)
(**b**)							
Alfouzan (2019) [39]	Total CS, N = 7235	SSI	152/7235 (2.1%)	Wound swab 112/148	Y/N for single bacteria	N.A	Reported MRSA, ESBL
De (2016) [35]	Total CS, N = 500	SSI	121/500 (24.2%)	Wound swab 128 isolates from 121 cases	Y/Y	*1. Acinetobacter spp.* *2. E.coli* *3. S. aureus*	No data on MRSA Extrapolated 3GCR
De Nardo (2013) [40]	Total CS, N = 467	SSI	225/467 (48.2%)	Wound swab 106/197 (53.8%)	Y/N	*1. CoNS* *2. S. aureus* *3. E. coli*	Reported MRSA, No data on 3GCR
Kifilie (2018) [34]	Women with an infection due to CS and episiotomy delivery, N = 107	SSI and episiotomy infections	/	Wound specimens 101/107	Y/Y	*1. S. aureus* *2. CoNS* *3. E. coli*	Extrapolated MRSA, 3GCR
Mpogoro (2014) [41]	Patients delivering by CS, N = 345	SSI	34/345 (9.9%)	Wound swab (25/34 swab, 72.3%) 22/25 bacterial isolates	Y/Y	*1. S. aureus* *2. K. pneumoniae* *3. E. coli*	Reported MRSA, ESBL
Njoku (2019) [27]	Total CS, N = 600	SSI	51/600 (8.5%)	Wound swab 47/51	Y/Y	*1. S. aureus* *2. K. pneumoniae* *3. E. coli*	Extrapolated MRSA, 3GCR
Onuzo (2022) [43]	Total CS, N = 474	SSI	61/474 (12.8%)	Wound swab 59/61 (96.7%) 54 bacterial isolates	Y/Y	*1. S. aureus* *2. E. coli* *3. K. pneumoniae, Proteus mirabilis, Pseudomonas spp.*	Extrapolated MRSA, No data on 3GCR
Salmanov (2021) (III) [37]	Total CS, N = 2326	SSI	342/2326 (14.7%)	Wound swab 534 bacterial strains from 342 women	Y/Y	*1. S. aureus* *2. E. coli*	Reported MRSA, ESBL
Salmanov (2020) (IV) [25]	Total deliveries with episiotomy procedures, N = 9213	Episiotomy infections	1628/9213 (17.7%)	Wound swab 2893 bacterial isolates from 1628 women	Y/Y	*1. E. coli* *2.Enterobacter spp.* *3. Streptococcus spp.*	Reported MRSA and ESBL + *Enterobacteriaceae*, Extrapolated *E.coli* and *Klebsiella spp.* 3GCR
Utami (2020) [30]	Total CS, N = 4809	SSI	15/4809 (0.3%)	Wound swab 15 bacterial isolates	Y/Y	*1.CoNS* *2. S. aureus/* *S. pneumoniae/ E. coli/K. pneumoniae*	No data on MRSA, Extrapolated 3GCR (cefotaxime instead of ceftriaxone)
Velin (2021) [44]	Total CS, N = 795	SSI	45/795 (5.7%)	Wound swab 44/45 (97.8%) 57 pathogens isolated	Y/Y	*1. CoNS* *2.Acinetobacter baumannii* *3.K. pneumoniae, Proteus spp., Enterobacter cloacae*	Extrapolated MRSA, 3GCR
Wekesa (2020) [26]	Women with SSI post CS, N = 109	SSI	/	Wound swab. 118 bacterial isolates in 109 women	Y/Y	*1. Klebsiella spp.* *2. S. aureus* *3. Enterococcus spp.*	Reported MRSA, Extrapolated 3GCR

*N.A.* not available, *N* number, *CS* caesarean section, *SSI* surgical site infection, *Y/N* yes/no, *CoNS* Coagulase Negative *Staphylococcus*, *MRSA* methicillin-resistant *Staphylococcus aureus*, *3GCR* 3rd generation cephalosporin resistant, *ESBL* extended-spectrum beta-lactamase

### Quality Assessment

Quality scores are described in Table 5. Seven articles (31.8%) [24–29, 43] fulfilled all the criteria and were considered high-quality. Other 7 papers (31.8%) [31, 36–40, 44] met 4 criteria, 5 papers (22.7%) [23, 33–35, 41] met 3 criteria and 3 (6.6%) [30, 32, 42] met 2.

**Table 5:**
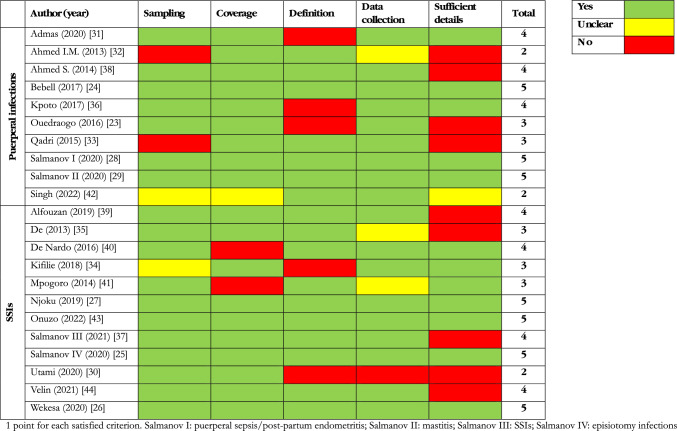
Quality assessment of studies included in the systematic review

The main reasons for poor quality were criterion 5, *i.e.,* poor characterization of the study population and setting (9/22, 40.9%), and criterion 3, *i.e.,* the lack of a standard definition for maternal infection (5/22, 27.3%). Regarding study population, in one paper [25] we defined criterion 5 as totally satisfied even if the population description did not include all the characteristics of the other studies. This paper reported episiotomy infections after vaginal delivery, and did not specify whether the delivery was performed in emergency or not, compared to the other articles on SSIs after CS.

### Frequency of Maternal Post-Partum Infections

#### Puerperal Infections Except for SSIs

The frequency of post-partum puerperal sepsis, according to the WHO definition, ranged from 1.4% in Burkina Faso [23] to 48.2% in Uganda [24] (Tables 2 and 3). The frequency of UTIs (14%) was reported in only one article from Uganda [24], as well as the frequency of post-partum mastitis, which was described in only one study conducted in Ukraine (22.6%) (Tables 2 and 3) [29]. All 10 articles on puerperal infections reported data on bacterial isolates and their antimicrobial susceptibility profile [23, 24, 28, 29, 31–33, 36, 38, 42], except for one paper, that did not describe good-quality microbiological information [42]. The most frequently isolated bacteria were *Escherichia coli* and *Staphylococcus aureus* (Table 3).

#### SSIs

Eight out of twelve studies on wound infections reported data on SSIs secondary to CS, one described episiotomy infections, and one described data on both (Tables 2 and 4).

The frequency of wound infections ranged from 0.3% in Indonesia [30] to 48.2% in Tanzania [40], whereas the only study reporting the frequency of episiotomy infections described a rate of 17.7% and was performed in Ukraine [25] (Table 4). Infections mostly occurred within 15 days from the incision (≤ 15 days [39], ≤ 7 days [35], < 9 days [40]). Women were followed up for 30 days after hospital discharge in 8 studies [26, 27, 35, 37, 39–41, 43], and for 10 and 11 days in the other two studies in Ukraine and Rwanda, respectively (Table 2b) [25, 44]. Two studies did not specify a follow-up period.

All 12 studies [25, 27, 34, 35, 37, 39–41, 43, 44] described data on bacterial isolates and antimicrobial susceptibility, except for 2 articles [39, 40], that directly reported information on MDR pathogens frequency. The most frequently bacterial isolates were *Escherichia coli, Klebsiella spp.,* and *Staphylococcus aureus* (Table 4).

### Frequency of Infections Caused by MDR Pathogens

Not all the studies reported data on MRSA and ESBL frequency. In such cases, we extrapolated this information from the bacterial in vitro susceptibility pattern for cefoxitin/oxacillin and ceftriaxone, respectively.

#### Frequency of Infections Caused by MRSA

A total of 17/22 studies [23, 25–29, 31–34, 37–41, 43, 44] reported data on MRSA infections. Supplementary Table 3 shows the frequencies and the diagnostic methods used for the detection of MRSA. We assessed the methicillin-resistance pattern from the in vitro non-susceptibility to cefoxitin/oxacillin of bacterial isolates in 6 studies [23, 27, 31, 34, 43, 44].

Among the 10 studies on puerperal infections, seven [23, 28, 29, 31–33, 38] described the prevalence of MRSA infections (Table 3, Suppl. Table 3). With regards to diagnostic methods, the methicillin-resistance profile of *S. aureus* was detected through MRSA strip colour test (N = 1) [32], Multiplex PCR assay for the assessment of *mecA* (N = 1) [38], VITEK-2 system (N = 1) [31], or through disk diffusion methods (N = 4) (Suppl. Table 3) [23, 29, 32, 33]. We extrapolated the methicillin resistance through the in vitro non-susceptibility profile to cefoxitin or oxacillin in 2 studies (Table 3) [23, 31].

Overall, the proportion of MRSA in puerperal infections ranged from 15.4% in Ukraine [28] to 83.7% in Sudan [32] (Suppl. Table 3). Interestingly, a study conducted in Sudan reported an extremely high rate of MRSA (41/49, 83.7%) among women delivering at home, therefore speculating a community acquisition of MRSA [32].

Among the 12 studies on SSIs, ten [25–27, 34, 37, 39–41, 43, 44] described the frequency of MRSA infections (Table 4, Suppl. Table 3). With regards to diagnostic methods, the methicillin resistance profile of *S. aureus* was detected by PCR testing for *mecA* gene (N = 1) [26], VITEK 2 system (N = 1) [44], or disk diffusion method (N = 6) [25, 34, 35, 37, 41, 43]. We extrapolated the methicillin resistance in 4 studies (Table 4) [27, 34, 43, 44]. In 2 studies the diagnostic method was not specified [27, 39].

Overall MRSA rate in SSIs ranged from 13.9% in Ukraine [37] to 91.2% in Uganda [26] and 100% in Rwanda [44], but in the latter sample size was very low (2 patients).

**Fig. 2:**
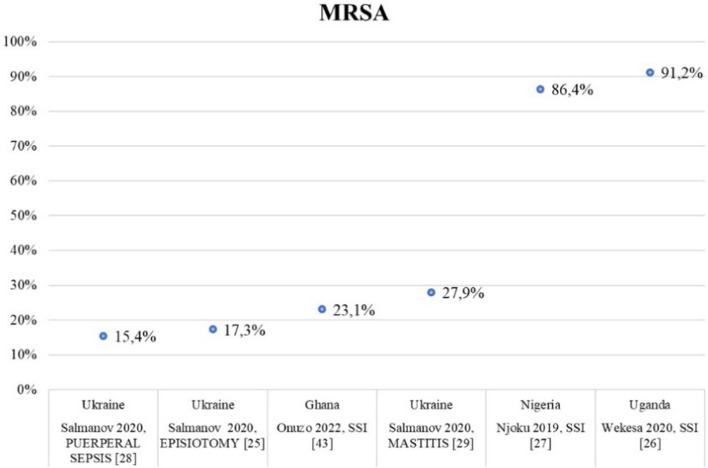
Burden of MRSA in all high-quality studies included in the systematic review

Figure 2 represents the frequency of MRSA strains reported in 6/7 high-quality studies [25–29, 43]: MRSA ranged from 9.8% in Ghana [43] to 91.2% in Uganda [26], with higher frequencies of infections among wound infections (4/6, 66.7%) compared to puerperal infections (2/6, 33.3%). Moreover, we found a higher rate of MRSA infections in African regions (Nigeria, Uganda) compared to Ukraine (Fig. 2).

#### Frequency of Infections Caused by ESBL-Producing Pathogens

A total of 19 articles [23–31, 33–39, 41, 42, 44] reported data on ESBL-producing *Enterobacterales* infections. Table 4 and Supplementary Table 4 show the frequencies and the diagnostic methods used for the detection of ESBL or 3GCR.

We used the data of 3GC resistance as suggestive of ESBL production in 12 studies, among which we used the in vitro susceptibility to ceftriaxone in all cases [23, 26–28, 31, 32, 34, 35, 37, 42, 44], except for one, in which we used cefotaxime susceptibility because ceftriaxone was not tested [31] (Suppl. Table 4).

Among the ten studies on puerperal infections, nine [23, 24, 28, 29, 31, 33, 36, 38, 42] reported the frequency of ESBL-producing *Enterobacterales* (Table 4 and Suppl. Table 4). ESBL production was assessed through the Multiplex PCR assay (N = 1) [38], synergy test between ceftazidime or cefotaxime and amoxicillin/clavulanic acid (N = 1) [24], VITEK-2 system (N = 1) [31], and disk diffusion methods (N = 4) [28, 29, 33, 42]. We extrapolated 3GCR of *Enterobacterales* through the in vitro non-susceptibility to ceftriaxone in 4 studies [23, 29, 31, 42]; in another study frequency of ESBL-producing *E. coli* was reported in the text, and we extrapolated it for *Enterobacterales* and *Klebsiella spp*. [38] (Suppl. Table 4).

Overall, the frequency of ESBL-producing *Enterobacterales* in puerperal infections ranged from 8% in India [33] to 82% in Uganda (UTIs) [24] (Suppl. Table 4): ESBL-producing *Escherichia coli* ranged from 25.8% in Ukraine [29] to 75.5% in Bangladesh [38], whereas *Klebsiella spp.* ranged from 16.7% in India [44] to 100% in Uganda [24].

Among the twelve studies on SSIs, ten [25–27, 30, 34, 35, 37, 39, 41, 44] reported the frequency of ESBL-producing *Enterobacterales* (Table 4, Suppl. Table 4). We extrapolated the ESBL production through the in vitro 3GCR in 7 papers [25–27, 30, 34, 35, 44]: in 6 studies we used ceftriaxone susceptibility [25–27, 34, 35, 44], and in one we used cefotaxime, since no data on ceftriaxone were available (Suppl. Table 4) [30].

Overall, the frequency of ESBL-producing *Enterobacterales* in SSIs ranged between 18.3% in Ukraine [37] and 95.2% in Uganda [26]: ESBL-producing *Escherichia coli* ranged from 6.2% in Kuwait [39] and 100% in Uganda [26], whereas *Klebsiella spp.* ranged from 6.2% in Kuwait [39] and 100% in Nigeria, Rwanda, and Indonesia (Suppl. Table 4) [27, 30, 44].

**Fig. 3:**
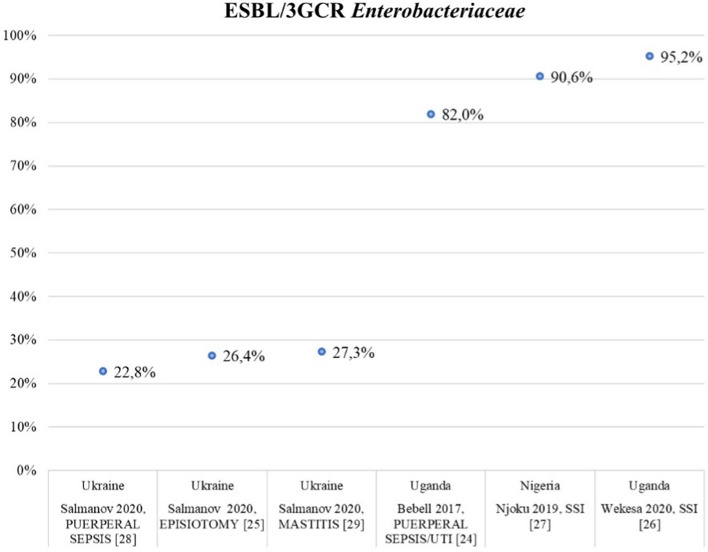
Burden of ESBL-producing/3GCR *Enterobacteriaceae* in all high-quality studies included in the systematic review

Figure 3 describes the frequency of ESBL-producing/3GCR *Enterobacterales* reported in 6/7 high-quality studies [24–29]. ESBL-producing *Enterobacterales* ranged from 22.8% in Ukraine [28] to 95.2% in Uganda [26]. We found a higher frequency of infections in African regions (Nigeria, Uganda) compared to Ukraine. Supplementary Figures 1 and 2 show the frequency of ESBL-producing/3GCR *E. coli* and *K. pneumoniae* in high-quality studies, respectively.

### Antibiotic Prophylaxis/Therapy Regimens

Nine articles, in particular those reporting data on infections secondary to CS, described different protocols on peripartum antibiotic prophylaxis or post-partum therapies in different countries.

In Mbarara Regional Referral Hospital, in Uganda, 802/875 (92%) women delivering by CS received a single dose of peri-operative antibiotic (ampicillin or ceftriaxone) within 30 min from skin incision, and 788/875 (90%) received a combination of intravenous (IV) ceftriaxone and metronidazole after CS for 3 days, followed by 5 days of oral cefixime [24]. Likewise, in Mulago Hospital in Kampala, Uganda, almost all patients undergoing CS received IV ceftriaxone and/or metronidazole either pre-, intra-, or post-surgery, as well did women with post-CS surgical site infections [26]. In Dodoma Regional Referral Hospital, Tanzania, even though only 10 women on total CS (10/467, 2.1%) received peri-operative antibiotic prophylaxis and only 2 received the dose 30–60 min before skin incision, a 3-day IV antibiotic course with ceftriaxone and metronidazole was prescribed in almost all women, followed by ampicillin/cloxacillin and metronidazole for at least another 5 days [40]. In another hospital in Tanzania, the Bugando Medical Center in Mwanza, almost all women (99.7%) delivering by CS received antibiotic prophylaxis with different timing before or after skin incision. The choice of antibiotic was based on the indication of CS and the surgeon’s preference, with 49.4% of women receiving ampicillin-based regimens [41]. Two retrospective cohort studies conducted in Ukraine to assess the prevalence of post-partum endometritis and SSI described the tendency to prescribe a post-partum combination of ceftriaxone and metronidazole in 90.9% and 83.7% of women delivering by CS, respectively [28, 37]. Nevertheless, in both cases, a high percentage of women, 86.7% and 95.9%, respectively, received peri-operative prophylaxis with beta-lactams [28, 37]. Another retrospective cohort study conducted in Ukraine to assess the rate of episiotomy infections in the puerperium described the habit to prescribe ceftriaxone and metronidazole post-partum in 89.9% of women after vaginal delivery [25]. Lastly, at the Lady Hardinge Medical College in New Delhi, India, antibiotics such as ampicillin, metronidazole, and gentamicin, are normally administered at the rupture of membranes or 30 min before CS [35].

Finally, there are countries where antibiotic prophylaxis is not administered pre-operatively nor intra-operatively, and where there are no standardized protocols for wound care, such as in Liberia [36], and countries where no routine but once-a-week operation theatre cleaning is performed, or whenever considered dirty, such as in Tanzania (in Mwanza), because of water shortage [41].

## Discussion

This systematic review aimed to assess the burden of infections due to MRSA and ESBL-producing *Enterobacterales* among women in the peri-/post-partum period in LMICs and to describe antimicrobials’ use in this setting. We focused on infections caused by MRSA and/or *Enterobacterales* ESBL-producing or resistant to 3GC because *Staphylococcus aureus* and *Enterobacterales* are the most common causative agents. Moreover, they respectively belong to the “high” and the “critical” priority list of antibiotic-resistant pathogens published in 2017 by WHO, and they are the two new AMR indicators within the target 3.d of SDGs (selected “sentinel” pathogens for BSI) [12, 15, 16].

We found 22 studies that met our inclusion criteria, including 14,804 total bacterial isolates from women from 12 different countries, mainly from the WHO African region. Seven out of the total 22 (31.8%) studies were considered high-quality.

MRSA and/or ESBL-producing *Enterobacterales* infection rates varied considerably between studies. When considering only high-quality studies, they were particularly high in Africa, both in women with puerperal and wound infections. However, studies were highly heterogeneous, and we did not deem it possible to compare them with statistical methods.

The studies included in this systematic review reported a wide range of post-partum infections frequency in LMICs, *i.e.*, 1.4–48.2% for puerperal sepsis and 0.3–48.2% for SSIs, probably due to differences in infection definition, surveillance and diagnostic methods, and patient population.

Puerperal sepsis is responsible for over 10% of maternal deaths worldwide and disproportionately occurs in LMICs [1]. However, although the frequency of deaths due to post-partum infections has considerably decreased in HICs (0.1–0.6/1000 births), these infections remain an important direct cause of maternal mortality in resource-limited settings [3, 5].

In the last decades, there has been an increasing rate of CS in both HICs and LMICs [46]. A recent systematic review and meta-analysis on the global incidence of SSIs secondary to CS described a higher pooled incidence of infections in LICs compared to HICs (9.94% *vs* 3.91%, 95% CI 8.38–11.63 and 3.51–4.32, respectively) [47]. Nowadays, CS is the most common operative procedure performed in sub-Saharan Africa and it is performed mainly in emergency [48]. Compared to HICs, CSs in sub-Saharan Africa are accompanied by higher morbidity and mortality rates, and higher rates of post-partum bacterial infections [48]. Another systematic review on maternal peri-partum infections by Woodd et al*.* reported a lower average frequency of SSIs secondary to CS (3.4%), extrapolated by 3 poor-quality studies performed in Africa, that however did not include perineal wound infections [21].

Regarding MDR pathogens, we focused on infections caused by MRSA and/or ESBL-producing *Enterobacterales* described in the 7 high-quality studies, performed in India, Uganda, Ukraine, and Ghana [24–29, 43]. MRSA frequency was 15.4% and 27.9% in endometritis and mastitis in Ukraine, respectively [28, 29], and 9.8%, 17.3%, and 91.2% among SSIs in Ghana, Ukraine, and Uganda, respectively [25, 26]. The frequency of ESBL-producing *Enterobacterales* infections ranged from 22.8% to 82% among puerperal infections [24, 28], and from 24.6% to 95.2% among SSIs [29, 33], in both cases in Ukraine and Uganda, respectively. According to these data, we found higher frequencies of MRSA and ESBL-producing *Enterobacterales* in Africa compared to Ukraine. MRSA and ESBL-producing *Enterobacterales* frequencies in Ukraine resemble those reported in Europe by the European CDC, *i.e.,* a median prevalence of 16.4% for MRSA, and 15.1% and 31.7% for 3GCR *E. coli* and *K. pneumoniae*, respectively [49]. Regarding sub-Saharan Africa, already in 2014 a high level of resistance to commonly used antibiotics, such as 3GCs, was reported among *Enterobacterales* isolates, with a prevalence up to 46.5% [50]. A more recent systematic review by Tadesse *et. al* described lower rates of MDR isolates in Africa in the overall population compared to the frequencies of our studies [51]. The author reported resistance to ceftriaxone, which is suggestive of ESBL production, in 593/2963 (20%) *Escherichia coli* isolates and in 545/1594 (34.2%) of *Klebsiella pneumoniae* isolates, and a median oxacillin-resistance rate of *Staphylococcus aureus* equal to 34.5% (IQR 12.6–68.2) on 2665 total *Staphylococcus aureus* isolates [51]. However, most studies included in this systematic review described community-acquired infections (40.3%). Conversely, we focused on maternal infections limited to the puerperium period and half of the studies we included in the review described SSIs, hence mainly hospital-acquired infections. Consequently, the results are not comparable.

The high prevalence of AMR in women in the peri-/post-partum period in resource-limited countries could be largely explained by the chronic misuse of antibiotics in these settings. Indeed, in our review, we found that the main prescribed regimen was a combination of ceftriaxone and metronidazole followed by a 5-day course of oral beta-lactam. This combination regimen prescribed in the postpartum period, without clinical indication, is not ideal, because the use of broad-spectrum antibiotics such as 3GC may induce the selection of bacterial strains to produce ESBL. Besides, in low-resource settings, common empiric antibiotic therapies for UTIs, endometritis, chorioamnionitis include ampicillin, ceftriaxone, ciprofloxacin, gentamicin, or a combination of them, antibiotics that may not be effective in a high ESBL-rate setting [52]. WHO has implemented guidelines trying to optimize the use and the prescription of antibiotics in resource-limited settings; however, overuse of antibiotics is still happening [51].

Improving our understanding of the epidemiology of infections due to MDR pathogens in low-income settings is critical to tackle AMR globally. Advocacy and funding for higher quality research and surveillance systems are essential to better understand the problem of AMR, to generate evidence, and to implement treatment protocols in each country according to local epidemiology [53]. Resources for diagnostic tests and microbiological methods for rapid detection of resistant strains may be needed too, together with the implementation of Antimicrobial Stewardship and IPC programs. Current WHO programs have been making strives on this in the last decade, but several low-income countries are falling behind [54].

This systematic review is the first to estimate the prevalence of post-partum infections due to MRSA or ESBL-producing *Enterobacterales* in LMICs. It also describes information on the misuse of antimicrobials and highlights the lack of standardized hygiene, infection control measures, and postnatal care protocols. A comprehensive search strategy of three databases was performed, including a manual review of reference lists of the most interesting papers and forward citation tracking to identify studies missed by database searching.

Nevertheless, our study had some limitations: we excluded potentially relevant articles due to the lack of data on antimicrobial susceptibility, we did not include papers written in all languages, and we had a narrow focus on MRSA and ESBL-producing *Enterobacterales*. Another important limitation is the quality of the included studies. Methods and designs were not always described exhaustively, the diagnostic methods of MDR pathogens were very different, and, in some cases, we had to extrapolate data from the bacteria in vitro susceptibility profile as they were not reported directly.

Crucially, almost all studies included in the systematic review were hospital-based, and they may not be representative of the general obstetric population. Most deliveries in LMICs are performed at home, under poor hygienic conditions, and by traditional birth attendants. As reported in a study performed in Sudan, the community acquisition of MDR pathogens, such as MRSA, can be very high. Moreover, since most post-partum infections appear after hospital discharge, in the absence of post-natal follow-up, infections can go undiagnosed and unreported [5].

## Conclusions

Misuse of antibiotics is contributing to AMR worldwide, particularly in low-resource settings. We have described a generally high frequency of post-partum infections caused by MRSA and ESBL-producing *Enterobacterales,* especially in the African region. However, frequencies varied substantially from setting to setting and only a few studies met quality standards.

There is an urgent need for high-quality and population-based studies to better describe the real burden of AMR in these countries and therefore tailor efforts according to local epidemiology. Furthermore, considering the alarmingly high burden of MDR pathogens we described in LMICs, preventive efforts, informed by precise data through the implementation/renovation of surveillance programs, should be a priority for clinicians and policymakers. It is only through a concerted, global effort to scale up advocacy, funding, higher quality research, and robust surveillance systems that we can gain a true insight into the huge threat of AMR among vulnerable populations in poor settings.

### Supplementary Information

Below is the link to the electronic supplementary material.

Supplementary file1 (DOCX 279 KB)

## Data Availability

No datasets were generated or analysed during the current study. The data can be requested to the corresponding author by email: Prof Nicola Coppola, nicola.coppola@unicampania.it.

## Abbreviations

AMR: Antimicrobial Resistance
BSI: Bloodstream infection
CDC: Center for Disease prevention and Control
eCDC: european Center for Disease prevention and Control
CS: Caesarean Section
ESBL: Extended-spectrum Beta-lactamase
3GCR: Third generation cephalosporin resistance
GLASS: Global Antimicrobial Resistance and Use Surveillance System
HIC: High-income countries
IPC: Infection Prevention and Control
IQR: Inter Quartile Range
IV: Intravenous
LMICs: Low-Middle Income Countries
MDR: Multidrug-resistant
MDRO: Multidrug-resistant Organism
MeSH: Medical Subject Headings
MRSA: Methicillin-resistant *Staphylococcus aureus*
SDGs: Sustainable Development Goals
SSI: Surgical Site Infection
UTI: Urinary Tract Infections
UI: Uncertainty Intervals
WHO: World Health Organization
