# Supplementary material for: Burden of Antimicrobial Resistance Among Women with Post-Partum Infections in Low-Middle Income Countries: A Systematic Review
Source: J Epidemiol Glob Health. 2024 Apr 17;14(2):274–90. doi: 10.1007/s44197-024-00222-8 (PMC11176113; doi:10.1007/s44197-024-00222-8)

# SUPPLEMENTARY MATERIAL

**Supplementary table 1:** Search Strategy and search terms

1. Maternal text AND sepsis (text OR MeSH)
2. Maternal infection (text OR MeSH)
3. Maternal complications with infection text OR MeSH
4. 1 OR 2 OR 3
5. Frequency text or MeSH
6. AMR text or MeSH
7. 4 AND 5 AND 6
8. LMICs or developing country (text)
9. 7 AND 8
10. Restricted to Human/2005 Current
11. 9 AND 10

**Maternal/pregnancy terms**

| **Texts** |  |
| --- | --- |
| matern* OR pregnan* OR childbirth OR intrapartum OR intra-partum OR postpartum OR post-partum OR peripartum OR peri-partum OR postnatal OR post-natal OR puerperal OR puerperium OR parturition OR obstetric OR labo?r OR partum OR deliver* OR perineal OR perineum OR caesarean OR perinatal OR peri-natal OR postpartal OR post-partal |  |

**Sepsis**

| **Texts** | **[MeSh]** |
| --- | --- |
| Sepsis OR septic OR septic?em* OR infect* OR organ failure OR bact?eriemia OR blood stream infection* | Sepsis/ OR sepsis syndrome*/ OR bacterial infection/OR bloodstream infection |

**Maternal Infection**

| **Texts** | **[MeSH]** |
| --- | --- |
| Endometritis OR endomyometritis OR endoparametritis OR amnionitis OR placentitis OR membranitis OR chorioamnionitis OR ((puerperal or childbed or postpartum or post-partum) adj (fever or pyrexia)) OR cervicitis OR puerperal peritonitis OR wound infect* OR surgical site infect* OR mastitis OR pelvic infection* | Chorioamnionitis/ OR pregnancy complications, infectious/ OR puerperal infection/OR mastitis/ OR perinatal infection/OR surgical wound infection |

**Maternal Complications with infection**

| **Texts** | **[MeSH]** |
| --- | --- |
| (Pregnan* OR obstetric OR postpartum OR post-partum or maternal) adj2 (complication* OR morbidit* OR outcomes OR near-miss) | Pregnancy complications/ OR obstetric labor complications/ or puerperal disorders/ |
| AND | |
| (sepsis OR septic OR fever OR infection* OR pyrexi*) |  |

**Prevalence/incidence/study**

| **Texts** | **[MeSH]** |
| --- | --- |
| (prevalence OR proportion OR percent* OR frequency OR incidence OR rate* OR ratio OR cohort OR longitudinal study OR follow-up study OR prospective study OR retrospective study OR cross-sectional OR intervention study OR trial OR community-based study OR population-based study OR observational study OR evaluat* OR audit OR epidemiology) | (Prevalence/ OR incidence/ OR epidemiology/ OR epidemiologic methods/ OR clinical studies as topic/ OR epidemiologic studies/) |
| NOT | |
| (case report* or comment or practice guideline* or editorial or consensus development conference or guideline* or conference abstract or legal case* or legislation or newspaper article or patient education handout or retracted publication or review* or meta-analysis or outbreak investigation*) |  |

**Antimicrobial resistance**

| **Texts** | **[MeSH]** |
| --- | --- |
| Antimicrobial resistan* OR antibiotic resistan* OR antibacterial resistan* OR multidrug resistan* OR drug resistan* OR drug-resistan* OR 3rd generation cephalosporin resistan* OR fluoroquinolone resistan* OR Carbapenem-resistan* OR bacterial resistan* OR microb* OR AMR OR antibiotic susceptibility OR antimicrobial susceptibility | Antimicrobial drug resistance/OR beta-lactam resistance/OR drug resistance/OR antibiotic resistance/OR drug resistance, bacterial/OR drug resistance, microbial/OR methicillin resistance/OR vancomycin resistance/OR antibiotic sensitivity/OR drug resistance, multiple, bacterial/ |

**Setting (LMICs in each database)**

| **Texts** | **[MeSH]** |
| --- | --- |
| Developing countr* OR LMIC* OR low resource setting* OR low-resource setting* | Developing countr*/ |

**RESULTS**:

- EMBASE: 2450 + 325 = 2775 papers
- Medline: 2086 +327 = 2413 papers
- Global Health: 2617 + 519 = 3136 papers

**Supplementary table 2.** PRISMA checklist for systematic reviews [17]

| **Section and Topic** | **Item #** | **Checklist item** | **Location where item is reported** |
| --- | --- | --- | --- |
| **TITLE** | | |  |
| Title | 1 | Identify the report as a systematic review. | Page 1, Line 2 |
| **ABSTRACT** | | |  |
| Abstract | 2 | See the PRISMA 2020 for Abstracts checklist. | Page 2, Line 27 |
| **INTRODUCTION** | | |  |
| Rationale | 3 | Describe the rationale for the review in the context of existing knowledge. | Pages 4-5, Lines 74-105 |
| Objectives | 4 | Provide an explicit statement of the objective(s) or question(s) the review addresses. | Page 5, Lines 106-111 |
| **METHODS** | | |  |
| Eligibility criteria | 5 | Specify the inclusion and exclusion criteria for the review and how studies were grouped for the syntheses. | Page 6-7, Lines 133-161 |
| Information sources | 6 | Specify all databases, registers, websites, organisations, reference lists and other sources searched or consulted to identify studies. Specify the date when each source was last searched or consulted. | Page 5 Lines 114-118 |
| Search strategy | 7 | Present the full search strategies for all databases, registers and websites, including any filters and limits used. | Page 5, Lines 119-125 |
| Selection process | 8 | Specify the methods used to decide whether a study met the inclusion criteria of the review, including how many reviewers screened each record and each report retrieved, whether they worked independently, and if applicable, details of automation tools used in the process. | Page 8, Lines 180-181 |
| Data collection process | 9 | Specify the methods used to collect data from reports, including how many reviewers collected data from each report, whether they worked independently, any processes for obtaining or confirming data from study investigators, and if applicable, details of automation tools used in the process. | Page 8, Line 182-190 |
| Data items | 10a | List and define all outcomes for which data were sought. Specify whether all results that were compatible with each outcome domain in each study were sought (e.g. for all measures, time points, analyses), and if not, the methods used to decide which results to collect. | Page 6, Lines 129-132 |
|  | 10b | List and define all other variables for which data were sought (e.g. participant and intervention characteristics, funding sources). Describe any assumptions made about any missing or unclear information. | Page 8, Lines 191-193 |
| Study risk of bias assessment | 11 | Specify the methods used to assess risk of bias in the included studies, including details of the tool(s) used, how many reviewers assessed each study and whether they worked independently, and if applicable, details of automation tools used in the process. | Pages 8-9, Lines 195-207, table 1 |
| Effect measures | 12 | Specify for each outcome the effect measure(s) (e.g. risk ratio, mean difference) used in the synthesis or presentation of results. | N.A. |
| Synthesis methods | 13a | Describe the processes used to decide which studies were eligible for each synthesis (e.g. tabulating the study intervention characteristics and comparing against the planned groups for each synthesis (item #5)). | Pages 6, Lines 137-141 |
|  | 13b | Describe any methods required to prepare the data for presentation or synthesis, such as handling of missing summary statistics, or data conversions. | N.A. |
|  | 13c | Describe any methods used to tabulate or visually display results of individual studies and syntheses. | N.A. |
|  | 13d | Describe any methods used to synthesize results and provide a rationale for the choice(s). If meta-analysis was performed, describe the model(s), method(s) to identify the presence and extent of statistical heterogeneity, and software package(s) used. | N.A. |
|  | 13e | Describe any methods used to explore possible causes of heterogeneity among study results (e.g. subgroup analysis, meta-regression). | N.A. |
|  | 13f | Describe any sensitivity analyses conducted to assess robustness of the synthesized results. | N.A. |
| Reporting bias assessment | 14 | Describe any methods used to assess risk of bias due to missing results in a synthesis (arising from reporting biases). | N.A. |
| Certainty assessment | 15 | Describe any methods used to assess certainty (or confidence) in the body of evidence for an outcome. | N.A. |
| **RESULTS** | | |  |
| Study selection | 16a | Describe the results of the search and selection process, from the number of records identified in the search to the number of studies included in the review, ideally using a flow diagram. | Page 9, Lines 209-213, fig.1 |
|  | 16b | Cite studies that might appear to meet the inclusion criteria, but which were excluded, and explain why they were excluded. | Page 9, Lines 213-220, fig.1 |
| Study characteristics | 17 | Cite each included study and present its characteristics. | Page 9-10 Lines 222-242, table 2, table 3a and 3b |
| Risk of bias in studies | 18 | Present assessments of risk of bias for each included study. | Page 10, Lines 244-252, table 4 |
| Results of individual studies | 19 | For all outcomes, present, for each study: (a) summary statistics for each group (where appropriate) and (b) an effect estimate and its precision (e.g. confidence/credible interval), ideally using structured tables or plots. | Pages 11-14, Lines 254-337; Tables 2, 3a and 3b |
| Results of syntheses | 20a | For each synthesis, briefly summarise the characteristics and risk of bias among contributing studies. | N.A. |
|  | 20b | Present results of all statistical syntheses conducted. If meta-analysis was done, present for each the summary estimate and its precision (e.g. confidence/credible interval) and measures of statistical heterogeneity. If comparing groups, describe the direction of the effect. | N.A. |
|  | 20c | Present results of all investigations of possible causes of heterogeneity among study results. | N.A. |
|  | 20d | Present results of all sensitivity analyses conducted to assess the robustness of the synthesized results. | N.A. |
| Reporting biases | 21 | Present assessments of risk of bias due to missing results (arising from reporting biases) for each synthesis assessed. | N.A. |
| Certainty of evidence | 22 | Present assessments of certainty (or confidence) in the body of evidence for each outcome assessed. | N.A. |
| **DISCUSSION** | | |  |
| Discussion | 23a | Provide a general interpretation of the results in the context of other evidence. | Pages 15-16, Lines 368-384 |
|  | 23b | Discuss any limitations of the evidence included in the review. | Page 15, Lines 380-381 |
|  | 23c | Discuss any limitations of the review processes used. | Page 18, Lines 443-454 |
|  | 23d | Discuss implications of the results for practice, policy, and future research. | Page 17, Lines 429-436 |
| **OTHER INFORMATION** | | |  |
| Registration and protocol | 24a | Provide registration information for the review, including register name and registration number, or state that the review was not registered. | N.A. |
|  | 24b | Indicate where the review protocol can be accessed, or state that a protocol was not prepared. | N.A. |
|  | 24c | Describe and explain any amendments to information provided at registration or in the protocol. | N.A. |
| Support | 25 | Describe sources of financial or non-financial support for the review, and the role of the funders or sponsors in the review. | Page 20, Line 485 |
| Competing interests | 26 | Declare any competing interests of review authors. | Page 20, Line 481 |
| Availability of data, code and other materials | 27 | Report which of the following are publicly available and where they can be found: template data collection forms; data extracted from included studies; data used for all analyses; analytic code; any other materials used in the review. | Page 20, Line 483 |

**Supplementary table 3.** MRSA infections frequency and relative diagnostic methods

|  | **Country, year** | **n/N tot** | **MRSA frequency (%)** | **Diagnostic method** |
| --- | --- | --- | --- | --- |
| **Puerperal infections** | Admas 2020, Ethiopia [31] | 10/19 | 52.6% | VITEK 2, cefoxitin |
|  | Ahmed M.I. 2013, Sudan [32] | 41/49 | 83.7% | MRSA strips test - disk diffusion, methicillin |
|  | Ahmed S. 2014, Bangladesh [38] | 7/27 | 25.9% | Multiplex PCR assay |
|  | Bebell 2017, Uganda [24] | No data | No data | No data |
|  | Qadri 2015, India [33] | 4/11 | 36% | MRSA detection not specified, disk diffusion |
|  | Kpoto 2017, Liberia [36] | No data | No data | No data |
|  | Ouedraogo 2016, Burkina Faso [23] | 7/18 | 40% | method not specified, cefoxitin |
|  | Salmanov 2020, Ukraine (I) [28] | N.A. | 15.4% | MRSA detection not specified, disk diffusion |
|  | Salmanov 2020, Ukraine (II) [29] | 39/140 | 27.9% | disk diffusion, oxacillin |
|  | Singh 2022, India [42] | No data | No data | No data |
| **SSIs** | Alfouzan 2019, Kuwait [39] | N.A. | 30.3% | Method not specified |
|  | De D 2013, India [35] | No data | No data | No data |
|  | De Nardo 2016, Tanzania [40] | 34/43 | 79% | disk diffusion, oxacillin - MRSA detection not specified |
|  | Kifilie 2017, Ethiopia [34] | 7/42 | 16.7% | disk diffusion |
|  | Mpogoro 2014, Tanzania [41] | 1/6 | 16.7% | disk diffusion, cefoxitin and oxacillin |
|  | Njoku 2019, Nigeria [27] | 19/22 | 86.4% | method not specified, cefoxitin |
|  | Onuzo 2022, Ghana [43] | 6/26 | 23.1% | disk diffusion, oxacillin |
|  | Salmanov 2021, Ukraine (III) [37] | N.A. | 13.9% | MRSA detection not specified - disk diffusion |
|  | Salmanov 2020, Ukraine (IV) [25] | 21/122 | 17.3% | disk diffusion, oxacillin |
|  | Utami 2020, Indonesia [30] | No data | No data | No data |
|  | Velin 2021, Rwanda [44] | 2/2 | 100 | VITEK 2, cefoxitin |
|  | Wekesa 2020, Uganda [26] | 31/34 | 91.2% | PCR testing for *mecA* gene - disk diffusion |

MRSA: methicillin-resistant *Staphylococcus aureus*, PCR: polymerase chain reaction

**Supplementary table 4.** ESBL-producing *Enterobacteriaceae* infections frequency and relative diagnostic methods

|  | **Country, survey year** | **n/N tot** | **ESBL frequency, n (%)** | **Method** |
| --- | --- | --- | --- | --- |
| **Puerperal infections** | Admas 2020, Ethiopia [31] | 14/27  11/18  3/7 | 51.9% *Enterobacteriaceae*  61.1% *E. coli*  42.9% *K. pneumoniae* | VITEK 2, 3GCR (ceftriaxone) |
|  | Ahmed M.I. 2013, Sudan [32] | No data | No data | ESBL strips test - disk diffusion |
|  | Ahmed S. 2014, Bangladesh [38] | 123/152  74/98  30/33 | 80.9% *Enterobacteriaceae*  75.5% *E. coli* (CTX-M)  90.9% *Klebsiella spp.* | Multiplex PCR assay (CTX-M, TEM, SHV) |
|  | Bebell 2017, Uganda [24] | 9/11 (UTIs)  4/6  3/3 | 82% *Enterobacteriaceae*  67% *E. coli*  100% *Klebsiella spp.* | Synergy test between ceftazidime or cefotaxime and amoxicillin/clavulanic |
|  | Qadri 2015, India [33] | 2/26 | 8% | ESBL detection not specified - disk diffusion |
|  | Kpoto 2017, Liberia [36] | N.A.  8/11 | N.A. *Enterobacteriaceae*  73% *E. coli* | Method not specified - 3GCR (ceftriaxone) |
|  | Ouedraogo 2016, Burkina Faso [23] | 12/30 | 40% *E. coli* | Method not specified - 3GCR (ceftriaxone)  ceftriaxone S not available for other *Enterobacteriaceae* |
|  | Salmanov 2020, Ukraine (I) [28] | N.A. | 22.8% *Enterobacteriaceae* | Disk diffusion - ESBL detection not specified |
|  | Salmanov 2020, Ukraine (II) [29] | 519/1904  238/922  88/416 | 27.3% *Enterobacteriaceae*  25.8% *E. coli*  21.1% *Klebsiella spp*. | Disk diffusion, 3GCR (ceftriaxone) |
|  | Singh 2022, India [42] | N.A.  3/18  N.A. | NA *Enterobacteriaceae*  16.7% *K. aerogenes*  N.A. *E. coli* | Disk diffusion, 3GCR (ceftriaxone) |
| **SSIs** | Alfouzan 2019, Kuwait [39] | N.A.  N.A.  N.A. | N.A. *Enterobacteriaceae*  6.2% *E. coli*  6.2% *K. pneumoniae* | ESBL detection not specified |
|  | De D 2013, India [35] | 14/47  5/24  6/19 | 29.8% *Enterobacteriaceae*  20.8% *E. coli*  33.3% *Klebsiella spp.* | Disk diffusion, 3GCR (ceftriaxone) |
|  | De Nardo 2016, Tanzania [40] | No data | No data | Disk diffusion |
|  | Kifilie 2017, Ethiopia [34] | 20/42  12/20  8/14 | 47.6% *Enterobacteriaceae*  60% *E. coli*  57.1% *K. pneumoniae* | disk diffusion, 3GCR (ceftriaxone) |
|  | Mpogoro 2014, Tanzania [41] | 2/9 | 22.2% *Enterobacteriaceae* | ESBL detection not specified, disk diffusion |
|  | Njoku 2019, Nigeria [27] | 29/32  10/13  19/19 | 90.6% *Enterobacteriaceae*  76.9% *E. coli*  100% *Klebsiella spp.* | Method not specified, 3GCR (ceftriaxone) |
|  | Onuzo 2022, Ghana [43] | No data | No data | No data |
|  | Salmanov 2021, Ukraine (III) [37] | N.A.  N.A.  N.A. | 18.3% ESBL+ *Enterobacteriaceae*  15.2% 3GCR E. coli  7.9% 3GCR K. pneumoniae | ESBL detection not specified  Disk diffusion, 3GCR |
|  | Salmanov 2020, Ukraine (IV) [25] | N.A.  396/1423  66/255 | 26.4% ESBL+ *Enterobacteriaceae*  27.8% 3GCR *E. coli*  26.1% 3GCR *Klebsiella spp*. | ESBL detection not specified  Disk diffusion, 3GCR (ceftriaxone) |
|  | Utami 2020, Indonesia [30] | N.A.  2/2 | N.A. *Enterobacteriaceae*  100% *K. pneumoniae* | No data on ceftriaxone susceptibility 🡪 used cefotaxime  N.B. E coli not tested for cephalosporin |
|  | Velin 2021, Rwanda [44] | N.A.  3/3 | N.A. *Enterobacteriaceae*  100% *K. pneumoniae* | VITEK2, 3GCR (ceftriaxone) |
|  | Wekesa 2020, Uganda [26] | 60/63  11/11  43/44 | 95.2% *Enterobacteriaceae*  100% *E. coli*  97.7 % *Klebsiella spp.* | Disk diffusion, 3GCR (ceftriaxone) |

ESBL: extended-spectrum beta-lactamase; 3GCR: 3^rd^ generation cephalosporin resistant; PCR: polymerase chain reaction

**Supplementary figure 1.** Frequency of ESBL-producing/3GCR *E. coli* in high quality studies
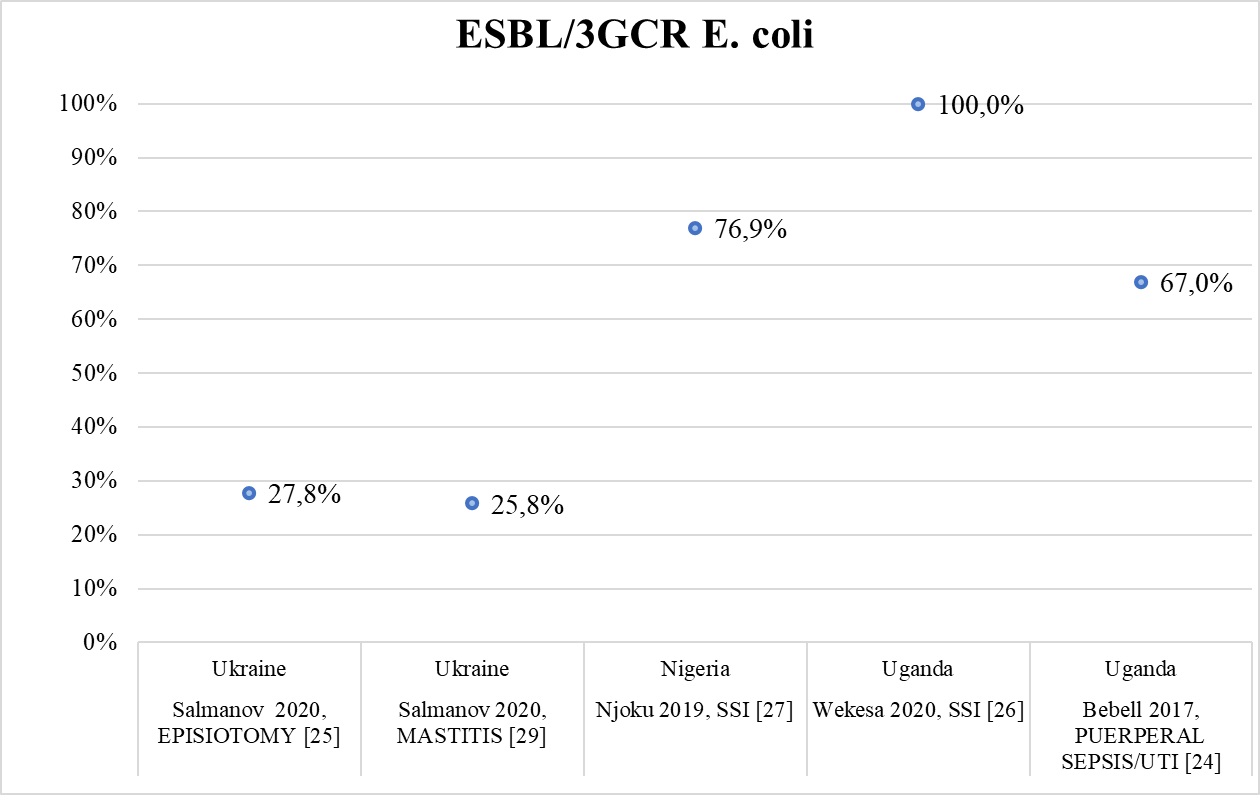


**Supplementary figure 2.** Frequency of ESBL-producing/3GCR *K. pneumoniae* in high quality studies


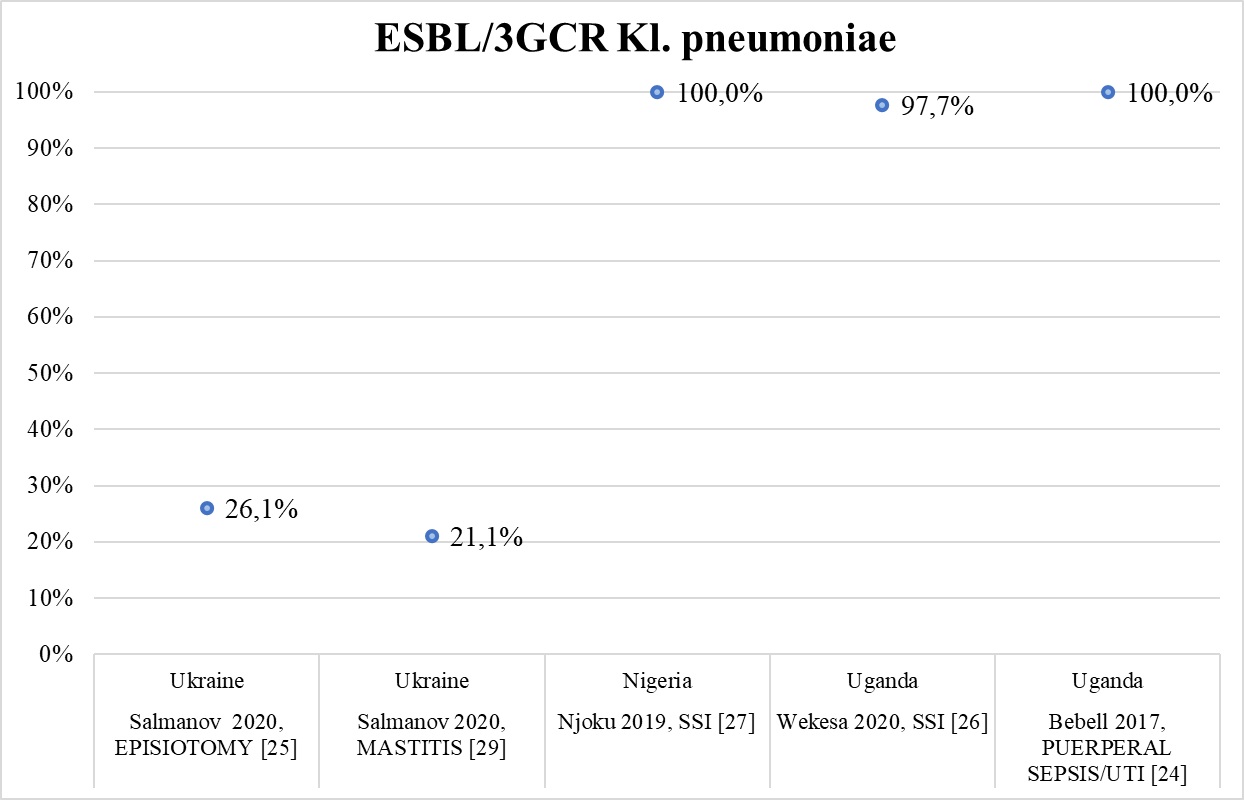

Supplement: Supplementary file 1 — Supplementary file1 (DOCX 279 KB) [file 44197_2024_222_MOESM1_ESM.docx]
